# 4-Carbethoxy-1-[4-(*N*,*N*-dimethylamino)benzoyl]thiosemicarbazide

**DOI:** 10.1107/S1600536810015576

**Published:** 2010-04-30

**Authors:** Hriday Bera, Anton V. Dolzhenko, Geok Kheng Tan, Lip Lin Koh, Wai Keung Chui

**Affiliations:** a Department of Pharmacy, Faculty of Science, National University of Singapore, 18 Science Drive 4, Singapore 117543, Singapore; b Department of Chemistry, Faculty of Science, National University of Singapore, 3 Science Drive 3, Singapore 117543, Singapore

## Abstract

The mol­ecular structure of the title compound, C_13_H_18_N_4_O_3_S, (systematic name: ethyl *N*-{2-[4-(dimethyl­amino)benzo­yl]hydrazinethio­carbon­yl}carbamate) is stabilized by intra­molecular N—H⋯O=C hydrogen bonding arranged in an *S*(6) graph-set motif. In the crystal, inversion dimers connected *via* inter­molecular N—H⋯S=C hydrogen bonds [*R*_2_^2^(8) graph-set motif] form sheets parallel to the (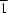
21) plane. Dimers are also formed by the mol­ecules *via* weak inter­molecular N—H⋯S=C hydrogen bonds [*R*_2_^2^(10) graph-set motif] connecting the sheets.

## Related literature

For examples of bioactive 1,4-diacyl substituted thio­semi­carbazides and their metal complexes, see: Angelusiu *et al.* (2009 ▶); Cunha *et al.* (2007 ▶); Qandil *et al.* (2006 ▶). For 4-aroyl-1-[4-(*N*,*N*-dimethyl­amino)benzo­yl]thio­semicarbazides as high affinity anion receptors, see: Liu & Jiang (2008 ▶). For the structures of related carbethoxy­thio­ureas, see: Dolzhenko *et al.* (2010**a* ▶,b* ▶). For the structures of related 1,4-diacyl thio­semicarbazides, see: Ali *et al.* (2004 ▶); Xue *et al.* (2006 ▶); Yamin & Yusof (2003 ▶); Yusof *et al.* (2003 ▶). For the graph-set analysis of hydrogen bonding, see: Bernstein *et al.* (1995 ▶).
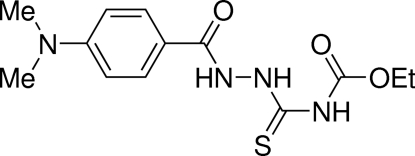


## Experimental

### 

#### Crystal data

C_13_H_18_N_4_O_3_S
*M* *_r_* = 310.37
Triclinic, 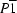

*a* = 7.876 (4) Å
*b* = 8.184 (4) Å
*c* = 12.086 (6) Å
α = 82.290 (12)°
β = 74.769 (11)°
γ = 84.469 (11)°
*V* = 743.3 (7) Å^3^
*Z* = 2
Mo *K*α radiation
μ = 0.23 mm^−1^
*T* = 100 K
0.24 × 0.10 × 0.08 mm

#### Data collection

Bruker SMART APEX CCD diffractometer
Absorption correction: multi-scan (*SADABS*; Sheldrick, 2001 ▶) *T*_min_ = 0.946, *T*_max_ = 0.982
5201 measured reflections
3379 independent reflections
2839 reflections with *I* > 2σ(*I*)
*R*_int_ = 0.031

#### Refinement

*R*[*F* ^2^ > 2σ(*F* ^2^)] = 0.081
*wR*(*F* ^2^) = 0.218
*S* = 1.18
3379 reflections
205 parameters
H atoms treated by a mixture of independent and constrained refinement
Δρ_max_ = 0.84 e Å^−3^
Δρ_min_ = −0.45 e Å^−3^

### 

Data collection: *SMART* (Bruker, 2001 ▶); cell refinement: *SAINT* (Bruker, 2001 ▶); data reduction: *SAINT*; program(s) used to solve structure: *SHELXS97* (Sheldrick, 2008 ▶); program(s) used to refine structure: *SHELXL97* (Sheldrick, 2008 ▶); molecular graphics: *SHELXTL* (Sheldrick, 2008 ▶); software used to prepare material for publication: *SHELXTL*.

## Supplementary Material

Crystal structure: contains datablocks I, global. DOI: 10.1107/S1600536810015576/ds2029sup1.cif

Structure factors: contains datablocks I. DOI: 10.1107/S1600536810015576/ds2029Isup2.hkl

Additional supplementary materials:  crystallographic information; 3D view; checkCIF report

## Figures and Tables

**Table 1:** Hydrogen-bond geometry (Å, °)

*D*—H⋯*A*	*D*—H	H⋯*A*	*D*⋯*A*	*D*—H⋯*A*
N1—H1*N*⋯S1^i^	0.82 (6)	2.53 (6)	3.342 (3)	173 (5)
N3—H3*N*⋯S1^ii^	0.80 (4)	2.64 (5)	3.385 (4)	156 (4)
N2—H2*N*⋯O2	0.86 (5)	2.02 (5)	2.653 (4)	130 (4)

Symmetry codes: (i) 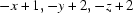; (ii) 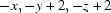.

**Fig. 1.**
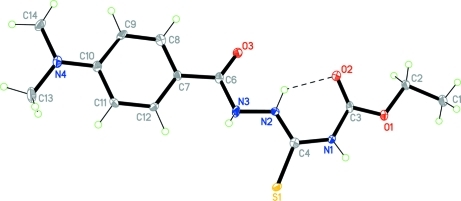
The molecular structure of 4-carbethoxy-1-[4-(N,N-dimethylamino)benzoyl]thiosemicarbazide, showing the atomic numbering scheme. Displacement ellipsoids are drawn at the 50% probability level.

**Fig. 2.**
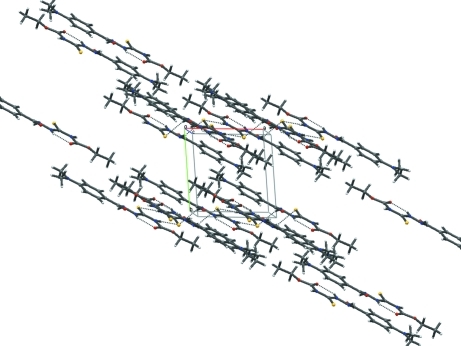
Crystal packing in the cell (view along axis c)

**Fig. 3.**
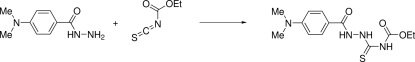
Synthesis of 4-carbethoxy-1-[4-(N,N-dimethylamino)benzoyl]thiosemicarbazide

## supplementary crystallographic information

### Comment

1,4-Diacyl substituted thiosemicarbazides and their metal complexes have been demonstrated to possess a potent antimicrobial activity (Angelusiu *et al.*, 2009; Cunha *et al.*, 2007; Qandil *et al.*, 2006). In continuation of our structural investigations of the carbethoxythioureas derivatives (Dolzhenko *et al.*, 2010*a,b*), we report herein molecular and crystal structure of 4-carbethoxy-1-[4-(*N*,*N*-dimethylamino)benzoyl]thiosemicarbazide (Figure 1 and 2). The compound is a structural analogue of 4-aroyl-1-[4-(*N*,*N*-dimethylamino)benzoyl]thiosemicarbazides reported recently as high affinity anion receptors (Liu & Jiang, 2008).

4-Carbethoxy-1-[4-(*N*,*N*-dimethylamino)benzoyl]thiosemicarbazide was prepared by nucleophilic addition of 4-(*N*,*N*-dimethylamino)benzhydrazide to ethoxycarbonyl isothiocyanate in DMF at room temperature (Figure 3).

The molecule of 4-carbethoxy-1-[4-(*N*,*N*-dimethylamino)benzoyl]thiosemicarbazide adopts similar to the previously reported (Ali *et al.*, 2004; Xue *et al.*, 2006; Yamin & Yusof, 2003;. Yusof *et al.*, 2003) for the related 1,4-diacyl substituted thiosemicarbazides configuration with the thiocarbonyl group pointed to the side opposite of the carbonyl groups. In the thiourea fragment, (*E*)- and (*Z*)-configurations observed across the C4—N1 and C4—N2 bonds, respectively. This configuration is stabilized by the strong intramolecular hydrogen bonding between N(2)—H and O2═C3 arranged in the *S*(6) graph-set motif (Bernstein *et al.*, 1995).

The thiourea C4—N2 bond is significantly shorter (1.315 (5) Å) than other C—N bonds of the molecule. The planarity of the molecule is affected by some twisting at the hydrazine N2—N3 fragment [—C4—N2—N3—C6— torsion angle is 166.5 (33)°].

In the crystal, the molecules form sheets parallel to the (121) plane (Figure 2). In the sheets, atom N1 of one molecule is involved in a intermolecular N(1)—H···S═C interaction with the thiocarbonyl atom S1 of adjacent molecule making pair with the *R*_2_^2^(8) graph-set motif. Dimmers are also formed by molecules *via* week intermolecular N(3)—H···S═C hydrogen bonds arranged in *R*_2_^2^(10)graph-set motifs connecting the sheets between each other.

### Experimental

To a fine suspension of 4-(*N*,*N*-dimethylamino)benzhydrazide in (0.54 g, 3.0 mmol) in anhydrous DMF (4 ml), ethoxycarbonyl isothiocyanate (0.37 ml, 3.3 mmol) was added. After stirring the mixture for 5 h at ambient temperature, cold water (50 ml) was added. The precipitated product was filtered, washed with cold water and recrystallized from toluene. Yield 0.83 g (89%), m.p. 201 °C (PhMe).

^1^H NMR (300 MHz, DMSO-*d_6_*): δ 1.26 (t, 3H, CH_3_, *J* 7.2 Hz), 2.99 (s, 6H, N(CH_3_)_2_), 4.21 (q, 2H, CH_2_, *J* 7.2 Hz), 6.74 (d, 2H, Ar, *J *8.7 Hz), 7.78 (d, 2H, Ar, *J* 8.7 Hz), 10.56 (s, 1H, NH), 11.34 (s, 1H, NH), 11.41 (s, 1H, NH).

^13^C NMR (75 MHz, DMSO-*d_6_*): δ 15.2, 40.1 (2 C), 62.7, 111.3 (2 C), 118.6, 129.6 (2 C), 153.1, 153.8, 165.0, 179.8.

### Refinement

All the H atoms attached to the carbon atoms were constrained in a riding motion approximation [0.95 Å for C_aryl_—H, 0.99 Å for methylenic protons and 0.98 Å for methyl groups; U_iso_(H) = 1.2U_eq_(C_aryl_), U_iso_(H) = 1.2U_eq_(C_methylenic_) and U_iso_(H) = 1.5U_eq_(C_methyl_)] while the N-bound H atoms were located in a difference map and refined freely.

### Crystal data

**Table:**

C_13_H_18_N_4_O_3_S	*Z* = 2
*M**_r_* = 310.37	*F*(000) = 328
Triclinic, *P*1	*D*_x_ = 1.387 Mg m^−^^3^
Hall symbol: -P 1	Melting point: 474 K
*a* = 7.876 (4) Å	Mo *K*α radiation, λ = 0.71073 Å
*b* = 8.184 (4) Å	Cell parameters from 644 reflections
*c* = 12.086 (6) Å	θ = 2.5–27.4°
α = 82.290 (12)°	µ = 0.23 mm^−^^1^
β = 74.769 (11)°	*T* = 100 K
γ = 84.469 (11)°	Rod, colourless
*V* = 743.3 (7) Å^3^	0.24 × 0.10 × 0.08 mm

### Data collection

**Table:**

Bruker SMART APEX CCD diffractometer	3379 independent reflections
Radiation source: fine-focus sealed tube	2839 reflections with *I* > 2σ(*I*)
graphite	*R*_int_ = 0.031
φ and ω scans	θ_max_ = 27.5°, θ_min_ = 1.8°
Absorption correction: multi-scan (*SADABS*; Sheldrick, 2001)	*h* = −9→10
*T*_min_ = 0.946, *T*_max_ = 0.982	*k* = −10→10
5201 measured reflections	*l* = −12→15

### Refinement

**Table:**

Refinement on *F*^2^	Primary atom site location: structure-invariant direct methods
Least-squares matrix: full	Secondary atom site location: difference Fourier map
*R*[*F*^2^ > 2σ(*F*^2^)] = 0.081	Hydrogen site location: inferred from neighbouring sites
*wR*(*F*^2^) = 0.218	H atoms treated by a mixture of independent and constrained refinement
*S* = 1.18	*w* = 1/[σ^2^(*F*_o_^2^) + (0.0987*P*)^2^ + 1.3625*P*] where *P* = (*F*_o_^2^ + 2*F*_c_^2^)/3
3379 reflections	(Δ/σ)_max_ < 0.001
205 parameters	Δρ_max_ = 0.84 e Å^−^^3^
0 restraints	Δρ_min_ = −0.45 e Å^−^^3^

### Special details

**Table:**

Geometry. All esds (except the esd in the dihedral angle between two l.s. planes) are estimated using the full covariance matrix. The cell esds are taken into account individually in the estimation of esds in distances, angles and torsion angles; correlations between esds in cell parameters are only used when they are defined by crystal symmetry. An approximate (isotropic) treatment of cell esds is used for estimating esds involving l.s. planes.
Refinement. Refinement of *F*^2^ against ALL reflections. The weighted *R*-factor *wR* and goodness of fit *S* are based on *F*^2^, conventional *R*-factors *R* are based on *F*, with *F* set to zero for negative *F*^2^. The threshold expression of *F*^2^ > 2σ(*F*^2^) is used only for calculating *R*-factors(gt) *etc*. and is not relevant to the choice of reflections for refinement. *R*-factors based on *F*^2^ are statistically about twice as large as those based on *F*, and *R*- factors based on ALL data will be even larger.

### Fractional atomic coordinates and isotropic or equivalent isotropic displacement parameters (Å^2^)

**Table:**

	*x*	*y*	*z*	*U*_iso_*/*U*_eq_	
S1	0.25106 (11)	0.89905 (11)	1.00357 (7)	0.0123 (3)	
O1	0.6976 (3)	1.2062 (3)	0.7276 (2)	0.0147 (6)	
O2	0.4814 (3)	1.1658 (3)	0.6437 (2)	0.0152 (6)	
O3	0.1155 (3)	0.9455 (3)	0.6162 (2)	0.0146 (6)	
N1	0.4741 (4)	1.0685 (4)	0.8327 (3)	0.0106 (6)	
H1N	0.534 (7)	1.072 (7)	0.878 (5)	0.037 (15)*	
N2	0.2296 (4)	0.9931 (4)	0.7882 (3)	0.0123 (6)	
H2N	0.262 (6)	1.043 (6)	0.719 (4)	0.017 (11)*	
N3	0.0781 (4)	0.9079 (4)	0.8084 (3)	0.0138 (7)	
H3N	0.005 (6)	0.926 (5)	0.866 (4)	0.011 (10)*	
N4	−0.6011 (4)	0.5754 (4)	0.7533 (3)	0.0218 (8)	
C1	0.9562 (5)	1.3511 (5)	0.6447 (3)	0.0151 (8)	
H1A	0.9156	1.4354	0.6979	0.023*	
H1B	1.0341	1.3997	0.5730	0.023*	
H1C	1.0207	1.2596	0.6803	0.023*	
C2	0.7995 (5)	1.2877 (5)	0.6182 (3)	0.0138 (7)	
H2A	0.7274	1.3800	0.5879	0.017*	
H2B	0.8385	1.2083	0.5601	0.017*	
C3	0.5467 (5)	1.1491 (4)	0.7259 (3)	0.0109 (7)	
C4	0.3184 (4)	0.9900 (4)	0.8667 (3)	0.0119 (7)	
C6	0.0248 (4)	0.8950 (4)	0.7109 (3)	0.0109 (7)	
C7	−0.1384 (4)	0.8092 (4)	0.7270 (3)	0.0108 (7)	
C8	−0.2115 (5)	0.8174 (4)	0.6328 (3)	0.0122 (7)	
H8	−0.1558	0.8778	0.5619	0.015*	
C9	−0.3619 (5)	0.7403 (5)	0.6401 (3)	0.0137 (7)	
H9	−0.4079	0.7473	0.5743	0.016*	
C10	−0.4491 (5)	0.6506 (4)	0.7447 (3)	0.0127 (7)	
C11	−0.3746 (5)	0.6417 (5)	0.8393 (3)	0.0145 (7)	
H11	−0.4295	0.5811	0.9104	0.017*	
C12	−0.2221 (5)	0.7201 (5)	0.8304 (3)	0.0124 (7)	
H12	−0.1742	0.7129	0.8955	0.015*	
C13	−0.6896 (5)	0.4845 (5)	0.8622 (4)	0.0228 (9)	
H13A	−0.6084	0.3962	0.8853	0.034*	
H13B	−0.7933	0.4363	0.8527	0.034*	
H13C	−0.7266	0.5600	0.9219	0.034*	
C14	−0.6706 (5)	0.5756 (5)	0.6535 (3)	0.0172 (8)	
H14A	−0.6649	0.6861	0.6103	0.026*	
H14B	−0.7935	0.5456	0.6789	0.026*	
H14C	−0.6004	0.4953	0.6037	0.026*	

### Atomic displacement parameters (Å^2^)

**Table:**

	*U*^11^	*U*^22^	*U*^33^	*U*^12^	*U*^13^	*U*^23^
S1	0.0110 (4)	0.0190 (5)	0.0080 (4)	−0.0039 (3)	−0.0034 (3)	−0.0013 (3)
O1	0.0134 (12)	0.0172 (13)	0.0144 (13)	−0.0080 (10)	−0.0040 (10)	0.0009 (10)
O2	0.0169 (13)	0.0166 (13)	0.0141 (13)	−0.0069 (10)	−0.0066 (10)	0.0008 (10)
O3	0.0172 (13)	0.0185 (13)	0.0092 (12)	−0.0073 (10)	−0.0032 (10)	−0.0018 (10)
N1	0.0119 (14)	0.0123 (14)	0.0092 (14)	−0.0051 (11)	−0.0045 (12)	−0.0004 (11)
N2	0.0122 (14)	0.0179 (15)	0.0072 (14)	−0.0068 (12)	−0.0028 (11)	0.0024 (12)
N3	0.0067 (13)	0.0220 (17)	0.0128 (16)	−0.0060 (12)	−0.0001 (12)	−0.0032 (13)
N4	0.0201 (17)	0.0303 (19)	0.0176 (17)	−0.0149 (15)	−0.0078 (14)	0.0034 (14)
C1	0.0134 (16)	0.0133 (17)	0.0183 (19)	−0.0044 (13)	−0.0034 (14)	0.0004 (14)
C2	0.0167 (17)	0.0143 (17)	0.0107 (17)	−0.0068 (14)	−0.0019 (14)	−0.0010 (13)
C3	0.0129 (16)	0.0051 (15)	0.0149 (17)	−0.0016 (12)	−0.0018 (13)	−0.0045 (12)
C4	0.0110 (16)	0.0113 (16)	0.0133 (17)	0.0005 (13)	−0.0031 (13)	−0.0024 (13)
C6	0.0109 (15)	0.0101 (16)	0.0122 (17)	−0.0015 (13)	−0.0016 (13)	−0.0050 (13)
C7	0.0091 (15)	0.0122 (16)	0.0104 (16)	−0.0028 (13)	−0.0004 (13)	−0.0012 (13)
C8	0.0130 (16)	0.0089 (16)	0.0142 (17)	0.0012 (13)	−0.0012 (13)	−0.0048 (13)
C9	0.0164 (17)	0.0180 (18)	0.0108 (17)	−0.0025 (14)	−0.0078 (14)	−0.0064 (14)
C10	0.0129 (16)	0.0119 (16)	0.0144 (18)	−0.0030 (13)	−0.0040 (14)	−0.0025 (13)
C11	0.0159 (17)	0.0163 (18)	0.0106 (17)	−0.0069 (14)	−0.0024 (14)	0.0028 (14)
C12	0.0111 (16)	0.0180 (18)	0.0096 (16)	−0.0035 (13)	−0.0049 (13)	−0.0004 (13)
C13	0.0180 (19)	0.025 (2)	0.026 (2)	−0.0121 (16)	−0.0032 (16)	−0.0026 (17)
C14	0.0189 (18)	0.0167 (18)	0.021 (2)	−0.0042 (14)	−0.0125 (15)	−0.0029 (15)

### Geometric parameters (Å, °)

**Table:**

S1—C4	1.690 (4)	C2—H2A	0.9900
O1—C3	1.325 (4)	C2—H2B	0.9900
O1—C2	1.465 (4)	C6—C7	1.479 (5)
O2—C3	1.219 (4)	C7—C12	1.394 (5)
O3—C6	1.221 (4)	C7—C8	1.396 (5)
N1—C3	1.374 (5)	C8—C9	1.372 (5)
N1—C4	1.379 (4)	C8—H8	0.9500
N1—H1N	0.82 (6)	C9—C10	1.414 (5)
N2—C4	1.315 (5)	C9—H9	0.9500
N2—N3	1.390 (4)	C10—C11	1.407 (5)
N2—H2N	0.86 (5)	C11—C12	1.389 (5)
N3—C6	1.371 (5)	C11—H11	0.9500
N3—H3N	0.80 (4)	C12—H12	0.9500
N4—C10	1.372 (5)	C13—H13A	0.9800
N4—C14	1.450 (5)	C13—H13B	0.9800
N4—C13	1.458 (5)	C13—H13C	0.9800
C1—C2	1.507 (5)	C14—H14A	0.9800
C1—H1A	0.9800	C14—H14B	0.9800
C1—H1B	0.9800	C14—H14C	0.9800
C1—H1C	0.9800		
			
C3—O1—C2	116.3 (3)	O3—C6—C7	123.2 (3)
C3—N1—C4	127.1 (3)	N3—C6—C7	116.7 (3)
C3—N1—H1N	112 (4)	C12—C7—C8	118.4 (3)
C4—N1—H1N	121 (4)	C12—C7—C6	123.7 (3)
C4—N2—N3	122.1 (3)	C8—C7—C6	117.9 (3)
C4—N2—H2N	124 (3)	C9—C8—C7	121.6 (3)
N3—N2—H2N	114 (3)	C9—C8—H8	119.2
C6—N3—N2	114.1 (3)	C7—C8—H8	119.2
C6—N3—H3N	119 (3)	C8—C9—C10	120.6 (3)
N2—N3—H3N	114 (3)	C8—C9—H9	119.7
C10—N4—C14	121.0 (3)	C10—C9—H9	119.7
C10—N4—C13	120.2 (3)	N4—C10—C11	121.3 (3)
C14—N4—C13	118.7 (3)	N4—C10—C9	121.0 (3)
C2—C1—H1A	109.5	C11—C10—C9	117.7 (3)
C2—C1—H1B	109.5	C12—C11—C10	121.0 (3)
H1A—C1—H1B	109.5	C12—C11—H11	119.5
C2—C1—H1C	109.5	C10—C11—H11	119.5
H1A—C1—H1C	109.5	C11—C12—C7	120.7 (3)
H1B—C1—H1C	109.5	C11—C12—H12	119.7
O1—C2—C1	106.0 (3)	C7—C12—H12	119.7
O1—C2—H2A	110.5	N4—C13—H13A	109.5
C1—C2—H2A	110.5	N4—C13—H13B	109.5
O1—C2—H2B	110.5	H13A—C13—H13B	109.5
C1—C2—H2B	110.5	N4—C13—H13C	109.5
H2A—C2—H2B	108.7	H13A—C13—H13C	109.5
O2—C3—O1	126.1 (3)	H13B—C13—H13C	109.5
O2—C3—N1	125.2 (3)	N4—C14—H14A	109.5
O1—C3—N1	108.7 (3)	N4—C14—H14B	109.5
N2—C4—N1	116.4 (3)	H14A—C14—H14B	109.5
N2—C4—S1	123.9 (3)	N4—C14—H14C	109.5
N1—C4—S1	119.7 (3)	H14A—C14—H14C	109.5
O3—C6—N3	120.0 (3)	H14B—C14—H14C	109.5
			
C4—N2—N3—C6	−166.5 (3)	N3—C6—C7—C8	169.8 (3)
C3—O1—C2—C1	176.1 (3)	C12—C7—C8—C9	0.0 (5)
C2—O1—C3—O2	−4.1 (5)	C6—C7—C8—C9	179.5 (3)
C2—O1—C3—N1	176.4 (3)	C7—C8—C9—C10	0.6 (5)
C4—N1—C3—O2	1.2 (6)	C14—N4—C10—C11	−175.8 (3)
C4—N1—C3—O1	−179.3 (3)	C13—N4—C10—C11	0.3 (6)
N3—N2—C4—N1	174.8 (3)	C14—N4—C10—C9	4.3 (6)
N3—N2—C4—S1	−6.0 (5)	C13—N4—C10—C9	−179.5 (4)
C3—N1—C4—N2	−0.2 (5)	C8—C9—C10—N4	178.9 (3)
C3—N1—C4—S1	−179.4 (3)	C8—C9—C10—C11	−0.9 (5)
N2—N3—C6—O3	5.2 (5)	N4—C10—C11—C12	−179.1 (4)
N2—N3—C6—C7	−178.5 (3)	C9—C10—C11—C12	0.8 (5)
O3—C6—C7—C12	165.6 (4)	C10—C11—C12—C7	−0.3 (6)
N3—C6—C7—C12	−10.6 (5)	C8—C7—C12—C11	−0.1 (5)
O3—C6—C7—C8	−13.9 (5)	C6—C7—C12—C11	−179.6 (3)

### Hydrogen-bond geometry (Å, °)

**Table:**

*D*—H···*A*	*D*—H	H···*A*	*D*···*A*	*D*—H···*A*
N1—H1N···S1^i^	0.82 (6)	2.53 (6)	3.342 (3)	173 (5)
N3—H3N···S1^ii^	0.80 (4)	2.64 (5)	3.385 (4)	156 (4)
N2—H2N···O2	0.86 (5)	2.02 (5)	2.653 (4)	130 (4)

Symmetry codes: (i) −*x*+1, −*y*+2, −*z*+2; (ii) −*x*, −*y*+2, −*z*+2.
